# Illness perception in functional neurological disorder: low illness coherence and personal control

**DOI:** 10.1136/bmjno-2024-000648

**Published:** 2024-05-23

**Authors:** Andreas Joos, Stoyan Popkirov, Claas Lahmann, Michael Jöbges, Christoph Herrmann, Philipp Maner, Kai Schörner, Gunnar Birke, Armin Hartmann

**Affiliations:** 1 Psychosomatic Medicine, University of Freiburg, Freiburg im Breisgau, Germany; 2 University of Essen, Essen, Germany; 3 Kliniken Schmieder Konstanz, Konstanz, Baden-Württemberg, Germany; 4 Kliniken Schmieder Gailingen, Gailingen, Baden-Württemberg, Germany

**Keywords:** CONVERSION DISORDER, FUNCTIONAL NEUROLOGICAL DISORDER

## Abstract

**Introduction:**

Illness perception refers to patients’ subjective representations and appraisals of somatic and mental symptoms. These are relevant for self-management and outcome. In clinical practice, patients with functional neurological disorder (FND) often encounter a fragmented biomedical attitude, which leaves them without clear concepts. In this context, illness perception is relevant.

**Methods:**

Illness perception was assessed in FND patients and compared with samples of psychosomatic patients (PSM) as well as poststroke patients (STR). The three samples (FND, n=87; PSM, n=97 and STR, n=92) were almost all in inpatient treatment or rehabilitation. Illness perception was assessed with the revised German version of the Illness Perception Questionnaire (IPQ-R). For assessments of correlations, depressive symptoms were tested with the Patient Health Questionnaire-9, dissociative and functional neurological symptoms by the German adaption of the Dissociative Experiences Scale and biopsychosocial complexity by the INTERMED Self-Assessment questionnaire.

**Results:**

Apart from the chronicity subscale, all dimensions of the IPQ-R differed between groups. FND patients perceived lower illness coherence and personal control than both other groups and attributed their illness more to chance than to behavioural risk factors. PSM patients had the strongest emotional representations. There were only few correlations with dissociative scores and biopsychosocial complexity.

**Conclusion:**

Illness perception is an important issue in patients with FND with particular emphasis on low illness coherence and personal control. Missing associations with biopsychosocial complexity suggest that subjective illness perception is an important complementary but separate issue, which likely influences therapeutic alliance and self-management in FND. Future studies should assess its influences on outcome.

**Trial registration number:**

DRKS00024685; German Clinical Trials Register; www.drks.de.

**WHAT IS ALREADY KNOWN ON THIS TOPIC**

- Illness perception, that is, patients’ subjective representations of somatic and mental symptoms seems of particular relevance in functional disorders.
- Illness perception influences self-management, seeking help from medical services and doctor–patient relationships.

**WHAT THIS STUDY ADDS**

- Patients with functional neurological disorder differ from other neurological patients (poststroke patients) with respect to severity of affective and somatic symptoms and illness perception.
- Patients with functional neurological disorder perceive lower illness coherence and personal control in comparison to poststroke patients and patients with other psychosomatic disorders.

**HOW THIS STUDY MIGHT AFFECT RESEARCH, PRACTICE OR POLICY**

- Illness perception in functional neurological disorder should be assessed in initial encounters, as it likely influences doctor–patient relationships.
- The influence of illness perception in functional neurological disorder on outcome should be assessed in future research.

## Introduction

Functional neurological disorder (FND) is commonly encountered in neurological practice and has high chronicity rates.^1 2^ In clinical practice, neurologists often find it difficult to treat and ‘manage’ these patients. Although the disorder is conceptualised as a biopsychosocial disorder nowadays, in daily practice, a rather fragmented and one-sided biomedical view and provision of healthcare prevails.^3 4^ Often, not only patients but also treating physicians (and other medical personnel) are somewhat ‘mystified’ by the somatic symptoms.^5^ These sociocultural circumstances of clinical practice, emphasised, for example, by Engel,^6^ are important and likely impact on illness perception in these patients.

The onset of somatic (as well as psychological) symptoms is followed by individual cognitive and emotional reactions in patients, which vary greatly.^7^ Patients build their own individual models of representations and appraisals of symptoms and illness, that is, illness representation. Leventhal developed a framework for examining individual perceptual, behavioural and cognitive processes when confronted with threats pertaining to the soma or psyche, that is, a *self-regulation model*.^8^ This has consequences for self-management, seeking help from medical services, adherence to suggested treatments, quality of life and outcome.^8^ This ‘Common-sense Model of Self-Regulation’ is dynamic and process oriented, that is, not static.^8 9^ Of note*, a mental health seeking model* builds on the self-regulation model and illness representations.^10^

Weinman and colleagues constructed a questionnaire focusing on the respective themes, that is, illness identity, consequences, timeline, that is, chronicity and control/cure.^7^ In 2002, the same group published an extended version, including cyclical timeline perceptions, illness coherence and emotional representations.^11^ The cyclical timeline assesses the perception of the variability of symptoms. Illness coherence pertains to the comprehension of symptoms as an entity to the patient with questions like, ‘I have a clear picture or understanding of my condition’ or ‘My illness is a mystery to me’ (reverse coding). Illness coherence includes metacognitive aspects and plays an important role in coping and the response to symptoms.^11^ The subjective perception of illness coherence is likely relevant in functional disorders, as patients often do not encounter clear-cut medical concepts associated with their somatic symptoms and suffering,^12^ which is likely due to the reductionistic biomedical view, described above; and occasionally rather one-sided nebulous and historical psychodynamic speculations. Naturally, cognitive and emotional aspects occur together and influence each other bidirectionally; therefore, the subscale emotional representation was included. Finally, the control subscale was differentiated into personal control and treatment control.^11^ In sum, perception and appraisal of bodily symptoms comprise complex multidimensional processes.^12^ The revised German version of the Illness Perception Questionnaire (IPQ-R) has been validated (see below).^13^

There have been some studies of illness perception in functional, that is, somatoform disorders.^12^ Weigel *et al* demonstrated that functional disorders themselves as well as functional disorders combined with comorbid somatic disorders showed unfavourable scores compared with patients with somatic disorders and healthy people in a population-based study using the Brief IPQ-R.^12^ Others demonstrated associations of illness perception with outcome in somatoform disorders as well as healthcare expenditure.^14 15^

In 2009, a prospective study focusing on FND was published using three items of the original IPQ^7^ focusing on chronicity and psychological factors.^16^ Illness beliefs, that is, expectation of non-recovery and non-attribution of somatic symptoms to psychological factors were independent predictors of outcome. Stone *et al* compared 102 patients with functional weakness with 43 patients with other neurological disorders causing weakness, like multiple sclerosis, with regards to illness perception.^5^ Two subscale domains were different: patients with FND scored lower on illness coherence and chronicity.^5^ One study compared two FND subgroups, functional limb weakness with functional seizures, using the IPQ-R,^11^ and in addition, this study compared patients with epileptic seizures with patients with weakness due to other neurological disorders (not FND); however, there were no formal comparisons between functional und non-functional disorders: patients with functional weakness had the perception of lower control, less consequences for themselves and family, and they attributed less psychological associations compared with functional seizure patients.^17^ Though there was no statistical formal comparison between functional und non-functional disorders in this study, the FND group as a whole showed lower illness coherence, that is, lower understanding of the condition. The same group investigated illness perception of the families focusing on differences between FND compared with other neurological disorders.^18^ From a comprehensive biopsychosocial perspective, systemic and family-based aspects are of importance with respect to aetiological and disorder maintaining factors as well as treatment-related themes.^12 19^ Family members of patients with FND attributed more often psychological explanations and a greater emotional impact than patients themselves; the study had not incorporated the coherence subscale.^18^

These findings reflect the importance of subjective attributions of patients with FND and the multidimensionality of the disorder. It is increasingly recognised, that it is of benefit to consider subjective assumptions and take time to discuss patients’ subjective illness beliefs^16^—as well as current (biopsychosocial) concepts and treatment options.^20^ Illness perception has received interest in various somatic disease entities, including stroke^21^ and mental disorders, like depression.^22 23^

In sum, on the one hand, there is limited data on illness perception in FND, which is of importance in particular at the beginning of diagnostic and treatment processes; on the other hand, there has been a surge of interest and scientific study of the disorder over the last two decades.^24^ As specific therapeutic concepts are rarely implemented in general neurological practice (apart from centres acquainted with the disorder),^24^ patients often remain unable to develop coherent illness models and respective coping strategies. This likely impacts on management and prognosis, including adherence and self-efficacy. The current study investigated a moderate sample size of strongly affected patients with FND. In order to put data of patients with FND into a perspective with other patient groups, we compared the FND sample with a poststroke population (STR) and inpatient and day clinic psychosomatic patients not suffering from FND (PSM). In a previous report concerning these samples, we had described high biopsychosocial complexity in FND.^4^

We hypothesised lower values of illness coherence in the FND sample due to high inexplicability of symptoms to the patients and previous reports.^5 12 17^ We expected higher scores of cyclic timeline due to the perception of higher symptom variability in patients with FND and PSM compared with STR. Due to high levels of affective burden in PSM and FND patients,^4 25^ we considered higher scores in emotional representation in these samples. Concordant with the data of patients with functional disorders in comparison to other medical conditions^12^ and data of depressive populations,^22^ we expected higher scores in the subscale consequences and lower scores in personal control in FND and PSM.

We did not formulate specific hypotheses with regards to causal subscales of the IPQ-R. In addition and exploratively, we correlated IPQ-R subscales, which differed significantly between groups, with scores of biopsychosocial complexity, dissociative symptom load and depression.

## Materials and methods

### Study design

It is registered at the German Clinical Trials Register (www.drks.de). Trained clinicians assessed patients eligible for the study. After informed consent, they completed the questionnaires.

### Participants

Most patients were in inpatient treatment: STR and most FND patients were in neurological rehabilitation (phase D, ie, not needing help with basic daily tasks). Patients with FND received psychotherapy during rehabilitation in addition.^26^ PSM patients were in inpatient or day clinic intensive psychotherapeutic treatment—which is quite unique in Germany and has a long tradition and is effective.^25 27^ Six per cent of patients with FND and 1% of PSM patients were seen as outpatients for further treatment planning.

Inclusion criteria were age ≥18 years, sufficient knowledge of the German language and meeting clinical diagnostic criteria for FND according to ICD-10, other psychosomatic disorders for the second group, that is, depression, anxiety, somatoform or eating disorders without FND and cerebral ischaemia or haemorrhage for STR. Exclusion criteria for all groups were psychotic disorders, substance abuse and relevant cognitive deficits.

Seventy-nine per cent of eligible patients participated.

### Instruments

The IPQ-R consists of several subscales, the first of which pertains to bodily symptoms (identity scale) and which is often not used in specific disorders like FND^17^; the other dimensions concern: consequences, personal control, treatment control, timeline acute/chronic, timeline cyclical, illness coherence and emotional representation. These are rated on 5-point Likert-type scales (strongly disagree, disagree, neither agree nor disagree, agree and strongly agree). These seven subscales comprise 38 items in the original version and 32 in the German version.^13^ The last dimension consists of 18 items with respect to possible causes, also rated on 5-point Likert-type scales; these are grouped into: psychological attributions, risk factors, immunity, accident or chance.^11^

The sum of values is divided by the number of items.^7^ Psychometric studies of the IPQ-R showed sound to good values.^11 13^ The revised German version of the IPQ-R has been validated confirming the factorial structure and Cronbach’s α coefficients in the satisfactory to good range, retest reliability and discriminative capability.^13^

Additionally, a German adaption of the Dissociative Experiences Scale (FDS),^28^ a depression questionnaire (PHQ-9), and the INTERMED Self-Assessment questionnaire (IMSA) for biopsychosocial complexity were used.^29^ With respect to severity of somatic and mental symptoms during the last week, Likert scales from 0 (none) to 10 (extreme) were used, and patients were asked to note the duration of somatic and/or mental symptoms.

### Statistics

Descriptive statistics characterised the sample in terms of demographic data and psychopathology. Analyses of variance, post hoc Tukey-Kramer and χ² assessed group differences, with p<0.05, two-sided. Group differences were controlled for significant group differences (ie, age, sex). Correlations between IPQ-R items, which differed between groups, and the PHQ-9, FDS and IMSA were using Spearman’s coefficient. Multiple correlative testing used Bonferroni corrections for significance thresholds.

## Results

Many patients with FND suffered from functional motor symptoms (48%) and a mixture of neurological symptoms (26%) (online supplemental table 1). PSM patients mostly suffered from depression (64%), anxiety (14%) and somatoform pain disorders (12%), and most STR had endured ischaemic cerebral disease (85%). Patients with FND had high affective comorbidity according to ICD-10 diagnoses (depression 39%, anxiety 21%) and somatoform pain disorder (15%; online supplemental table 1); posttraumatic stress disorder occurred in 7%. Strong affective comorbidity is also reflected by high PHQ-9 scores (table 1).

10.1136/bmjno-2024-000648.supp1 Supplementary data

**Table 1:** Sociodemographic data and psychopathology

	FND n=87	PSM n=97	STR n=92	ANOVA (df; F; p)*	Tukey-Kramer
Age (years)	44.8 (15.2)	45.9 (14.3)	56.6 (10.5)	2,273;21.6; 0.0001	STR > (FND, PSM)
Women (%)	75	56	59	χ²†= 8.1; p=0.02	
Living with partner (%)	28	40	61	χ²†= 21.0; p<0.0001	
Duration of symptoms (months)	52.1 (70.0)	23.6 (31.8)	15.8 (28.4)	2,238;12.6; 0.0001	FND > PSM > STR
Severity somatic symptoms†	6.7 (2.1)	5.4 (2.7)	4.1 (2.3)	2,272; 25.6; <0.0001	FND > PSM > STR
Severity mental symptoms†	5.8 (2.9)	6.8 (2.3)	3.9 (2.7)	2,272; 30.5; <0.0001	PSM > FND > STR
FDS_sum_	19.6 (15.1)	15.4 (13.8)	7.3 (6.8)	2,273; 22.8; <0.0001	(FND, PSM) > STR
PHQ-9	12.4 (6.6)	13.1 (5.8)	7.6 (5.4)	2,267; 23.1; <0.0001	(FND, PSM) > STR
IMSA_sum_	23.3 (8.2)	23.1 (7.4)	12.3 (6.7)	2,270; 65.2; <0.0001	(FND, PSM) > STR

Numbers reported are means and SD.

*If not otherwise specified.

†Scale 0 (none)–10 (extreme) over the last week.

FDS, Dissociative Experiences Scale; FND, functional neurological disorder patients; IMSA, INTERMED Self-Assessment questionnaire; PHQ-9, Patient Health Questionnaire-9; PSM, psychosomatic medicine patients; STR, stroke patients.

STR were older, more often living with a partner and showed lower scores of depression, dissociation and biopsychosocial illness complexity than both other groups (table 1). FND included more women and showed the highest scores of somatic symptoms and longest disease duration (table 1).

Apart from timeline/chronicity, six of the seven main subscales and the four causal subscales showed group differences (table 2). Patients with FND reported lower illness coherence and personal control than both other samples and attributed their illness more to chance than to behavioural risk factors. PSM patients had strongest emotional representations and highest psychological attribution of illness. Consequences and timeline cyclical were higher in FND and PSM compared with STR, and treatment control was lower in FND compared with STR. Differences remained significant, when controlled for age and sex.

**Table 2:** Illness perception

	FND n=87	PSM n=97	STR n=92	ANOVA (df; F; p)	Tukey-Kramer
IPQ scales					
Chronicity	3.7 (0.8)	3.7 (0.7)	3.5 (0.9)	2,273; 2.1; 0.12	
Cyclical timeline	3.2 (0.9)	3.4 (0.8)	2.4 (0.9)	2,273; 39.0; <0.0001	(FND, PSM) > STR
Consequences	3.8 (0.8)	3.9 (0.6)	3.4 (0.9)	2,273; 15.6; <0.0001	(FND, PSM) > STR
Personal control	2.9 (0.9)	3.5 (0.8)	3.5 (0.9)	2,273; 13.0; <0.0001	(PSM, STR) > FND
Treatment control	3.2 (0.8)	3.4 (0.7)	3.5 (0.7)	2,273; 4.9; 0.009	STR>FND
Illness coherence	2.8 (1.1)	3.5 (1.0)	3.6 (0.9)	2,273; 18.0; <0.0001	(PSM, STR) > FND
Emotional representation	3.4 (1.1)	3.8 (0.8)	3.1 (1.0)	2,273; 14.0; <0.0001	PSM>FND > STR
IPQ causes					
Psychological causes	2.8 (1.1)	3.7 (0.7)	2.5 (0.9)	2,261; 45.4; <0.0001	PSM > (FND, STR)
Risk factors	2.0 (0.7)	2.4 (0.6)	2.4 (0.6)	2,265; 9.0; 0.0002	(PSM, STR) > FND
Immunological causes	2.1 (0.9)	1.8 (0.8)	1.9 (0.8)	2,264; 3.2; 0.04	FND>PSM
Chance causes	2.7 (1.1)	2.0 (1.0)	2.2 (0.8)	2,268; 9.8; <0.0001	FND > (PSM, STR)

Numbers reported are means and SD.

FND, functional neurological disorder patients; IPQ, Illness perception Questionnaire; PSM, psychosomatic medicine patients; STR, stroke patients.

There were no strong correlations between IPQ subscales and self-reported symptom scores of depression, anxiety and biopsychosocial complexity apart from weak to moderate correlations for emotional representation and consequences across the samples (table 3). Otherwise, no clear sample-specific pattern of associations emerged (besides, correlations of STR were all within low overall scores, ie, representing floor effects.)

**Table 3:** Correlations IPQ-R and symptom scales and biopsychosocial complexity

		IPQcycletime	IPQperscontr	IPQcurecontr	IPQillncoher	IPQemorepr	IPQconsequ
FND	PHQ-9	**0.45**				**0.50**	0.35
	FDS_sum_	0.34				0.28	0.28
	IMSA_sum_	0.30		−0.22	−0.22	**0.38**	**0.40**
PSM	PHQ-9			**−0.34**		**0.37**	**0.45**
	FDS_sum_	0.25					
	IMSA_sum_			−0.29	−0.24	0.29	**0.35**
STR	PHQ-9	0.25	−0.27	−0.28	−0.31	**0.59**	**0.55**
	FDS_sum_	**0.41**	−0.29		−0.25	**0.41**	**0.55**
	IMSA_sum_	**0.35**				**0.35**	**0.45**

Weakened numbers: p<0.05, bold letters and grey boxes: Bonferroni corrected (p<0.0009).

FDS, German adaption of the Dissociative Experiences Scale; FND, functional neurological disorder patients; IMSA, INTERMED Self-Assessment questionnaire; IPQconsequ, IPQ consequences; IPQcurecontr, IPQ cure/treatment control; IPQcycletime, IPQ cyclical timeline; IPQemorepres, IPQ emotional representation; IPQillncoher, IPQ illness coherence; IPQperscontr, IPQ personal control; PHQ-9, Patient Health Questionnaire-9; PSM, psychosomatic medicine patients; STR, stroke patients.

## Discussion

This study assessed illness perception with the validated IPQ-R in FND in comparison to PSM and STR. Most dimensions of the IPQ-R differed between the three groups, in particular between STR and FND—apart from the scale timeline, that is, chronicity. This is understandable, as patients in all three samples were strongly affected, including long disease duration and needed inpatient treatment. In a previous study comparing FND patients with other neurologic patients, chronicity was lower in FND^5^; likely, our patient group was more strongly affected, reflected by the fact that most were inpatients and suffered for more than 4 years in the mean.

In the context of the patients’ perception of a chronic disorder, it is important to communicate to the patients the principal reversibility and functional nature of the disorder. In fact, psychoeducation is an important part throughout the treatment of FND.^20 24^

Consistent with our hypothesis, PSM and FND patients showed higher cyclic timeline, that is, the perception of the variability of symptoms, which seems rational, as symptoms show higher variability over time compared with STR. From a psychological point of view, this represents an important issue of coping in these patient groups, as often there is an ‘immanent/potential health threat’, meaning that somatic and/or mental symptoms have the potential to arise anew or deteriorate, often without clear (conscious) precursors. Naturally, sometimes there are psychosocial strains; this is, however, not always the case from a clinical standpoint and according to studies in FND.^30^ This unpredictability is an issue in the management and psychotherapeutic treatment of these patients. This is likely also reflected by higher scores of PSM and FND patients in the subscale consequences as well in lower personal control in FND, consistent with our hypotheses. Furthermore, patients with FND scored lower in treatment control than STR. In addition to unpredictability of symptoms, this might also reflect that patients with FND often do not encounter clear concepts of the disorder and treatment options (see the Introduction section).

Emotional representation was particularly high in PSM, and FND had higher scores than STR; this reflects the emotional burden of the former two disorders, as we had assumed. This is also demonstrated by high depression scores, which correlated with emotional representation in all three groups. This correlation was observed in other studies, too.^22^

With regard to our hypothesis, illness coherence was lower in patients with FND compared with the other samples. This is a similar to the findings of Stone *et al* with respect to FND.^5^ From a psychological perspective, there is a basic need in human nature for safety and comprehension,^31^ which implies understanding and having a coherent concept of somatic and mental symptoms, which, particularly at the onset of illness, represent acute threats. Bodily symptoms which cannot be understood by patients and medical personnel leave much uncertainty. This non-understanding likely induces various emotional reactions, and it probably contributes to symptom and illness maintenance.^32^

Schäfer *et al* could demonstrate that the sense of coherence, which is a key component to Antonovsky’s theory of salutogenesis, was negatively correlated with symptom severity at the end of psychosomatic rehabilitation, that is, it proved to be a predictor of symptom change.^31^ It should be noted that although illness coherence is closely related to the sense of coherence, the latter has a wider scope of meaning and comprises three components^33^: comprehensibility, which is most closely related to illness coherence; manageability, closely related to the sense of personal control and meaningfulness, which is a more existential philosophical aspect related to Frankl’s psychotherapeutic assumptions of individual searches for the meaning of life.^34^ Erikson and Lindström emphasise the relationships of the sense of coherence and health and quality of life as well as its embeddedness in sociocultural themes and the biopsychosocial context.^33 35^

With respect to FND, there is still a primarily biomedical perspective in the medical community, and patients with FND do often not receive clear-cut diagnostic labels and biopsychosocial perspectives of the disorder.^3 4 24^ Furthermore, patients still often find themselves in between the fields of psychiatry and neurology, and FND does not have a ‘home within healthcare systems’, as Finkelstein *et al* put it.^24^ Regardless of precise causal grounds of the disease, the lack of coherent concepts likely perpetuates and maintains the disorder, which often shows chronicity.^16 36^ In clinical practice, patients often report being puzzled by explanations that there is ‘nothing wrong’ with them. For example, in group therapy and psychoeducation settings, patients with FND are thankful to hear of current multidimensional concepts of the disorder and share their experiences with others.^26^ In this context, educational aspects of medical personnel (including students in the various professions) to become acquainted with functional disorders and learn from each other in multidisciplinary teams are essential.^24^ McLaren *et al* recently pointed out, that self-identification, that is, that symptoms are understood as a disorder, is relevant for help-seeking behaviour in depressive illness, in addition to openness for a biopsychosocial model.^10^ Balint stressed the importance of initial contacts with patients with somatoform disorders in general practice.^37^

Similar to other studies in the field,^5 18^ patients with FND related fewer psychological causes to their symptoms compared with the other patient groups. They saw causes rather by chance.

Bear *et al* 38 formulate the importance of illness perception (in the context of affective disorders) concisely: ‘Illness perceptions are shaped by a person’s knowledge and experience and the discourse of those close to them, and therefore tend to vary between individuals, as well as fluctuate over time and with new information (eg, new diagnostic information) and experiences’. And further: ‘…as these representations guide attitudes towards mental health services, help-seeking, coping, self-management and treatment adherence’. One might add that in functional disorders, patients particularly benefit from early explanations of the nature and concepts of the symptoms, that is, psychoeducation, in addition to listening to their subjective assumptions.^39^ These interventions strengthen the doctor–patient relationship in clinical encounters and the therapeutic alliance in psychotherapy,^9^ and they likely increase illness coherence and hence adherence to therapy. These issues are also central to the biopsychosocial model,^6^ and exploration of patients’ illness perceptions is likely a key starting point in integrated care; the participatory nature of treatment planning has recently been emphasised, in particular, as there is no ‘one size fits all’ approach particularly in functional disorders.^24^ With respect to FND, it seems important to note that there already are well-described integrative treatment concepts.^1 24^ Besides, Balint had already focused on the multidimensionality of patients’ illness perception and complex patient–doctor interactions in somatoform/functional disorders with respect to cognitive and emotional factors encompassing attachment and transference (and the dynamic unconscious) in his seminal research,^37^ which has recently been re-emphasised.^19^ Balint stressed the importance of the initial encounters of the patient and medical personnel, when internal representations and appraisals of symptoms are yet not ‘organised’, as he called it, in functional disorders.^37^

There were few significant correlations of IPQ-R scores with measures of depressive symptoms, biopsychosocial complexity or dissociative symptom load, apart from emotional representation and consequences, which is reasonable, as these represent emotional burden and consequences of the (often chronic) diseases. Also, the IMSA assesses expected strains in the future,^29^ which likely reflects the association in all groups with these two IPQ-R subscales. However, there were no correlations with illness coherence or control dimensions in FND. These results emphasise that illness perception should be regarded as a valuable complementary tool in addition to a biopsychosocial formulation in FND.^40^

In sum, illness perception is an important issue in patients with FND. In particular, low illness coherence and personal control are likely important for (self-) management and outcome. Low illness coherence probably reflects insufficient communication of current integrative biopsychosocial concepts of the disorder in clinical practice, which itself impacts on the doctor–patient relationship. Subjective illness perception seems an important complementary issue in the assessment of patients with FND in particular at initial medical encounters.

### Limitations

The sample size was moderate, though not large enough to differentiate subgroups of FND or PSM patients, respectively; furthermore, neurological disorders with more phasic or recurrent courses like multiple sclerosis might be another neurological disorder to compare with. Previous treatments and medical encounters might influence illness perception (not only to the better but also to the worse, considering the often disappointing experiences of patients, as described above). We did not collect such data systematically, and this should be considered in future studies. Finally, qualitative studies of illness perception, that is, interviews, would likely add information—which can influence treatment planning including psychological interventions.

## Data Availability

Data are available upon reasonable request.
