# Supplementary material for: Illness perception in functional neurological disorder: low illness coherence and personal control
Source: BMJ Neurol Open. 2024 May 23;6(1):e000648. doi: 10.1136/bmjno-2024-000648 (PMC11116876; doi:10.1136/bmjno-2024-000648)
Supplement: Supplementary data [file bmjno-2024-000648supp001.pdf]

Suppl. Figure 1

Main Diagnoses

FND

ICD-10	Numbers (%)
F44.4 (Functional movement disorder)	42 (48)
F44.5 (Functional seizure disorder)	7 (8)
F44.6 (Functional sensory disorders)	10 (11)
F44.7 (Mixed functional disorder)	23 (26)
F44.7 (Functional neurological disorder, not specified)	5 (6)
sum	87 (100%)

PSM

ICD-10	Numbers (%)
F32/33 (Depressive disorders)	62 (64)
F45.4 (Somatoform pain disorder)	12 (12)
F45.0/1/3 (Somatoform disorders)	5 (5)
F41 (Anxiety disorders)	14 (14)
F43.1 (PTBS)	1 (1)
F45.2 (Hypochondriasis)	1 (1)
F50.0 (Anorexia nervosa)	1 (1)
F60.3 (Personality disorder)	1 (1)
sum	97 (100%)

STR

ICD-10	Numbers (%)
I60	4 (4)
I61/2/69.1	10 (11)
I63/64/67/69.3/4/8	78 (85)
sum	92 (100%)

Secondary F- Diagnoses

FND

ICD-10	Numbers
F06/07	4
F31	2
F32/33/38	34
F41	18
F43 (F43.1)	15 (6)
F45 (F45.4)	18 (13)
F50	2
F60	3
F84/90	4

PSM

ICD-10	Numbers (%)
F32/33/34	29
F40/41	5
F42	2
F43.1	6
F45.4	10
F45.0/1/3	15
F50	8
F60/66/90	3

STR

ICD-10	Numbers
F06/7 (F06.7)	19 (15)
F32/3	8
F41/3	4
